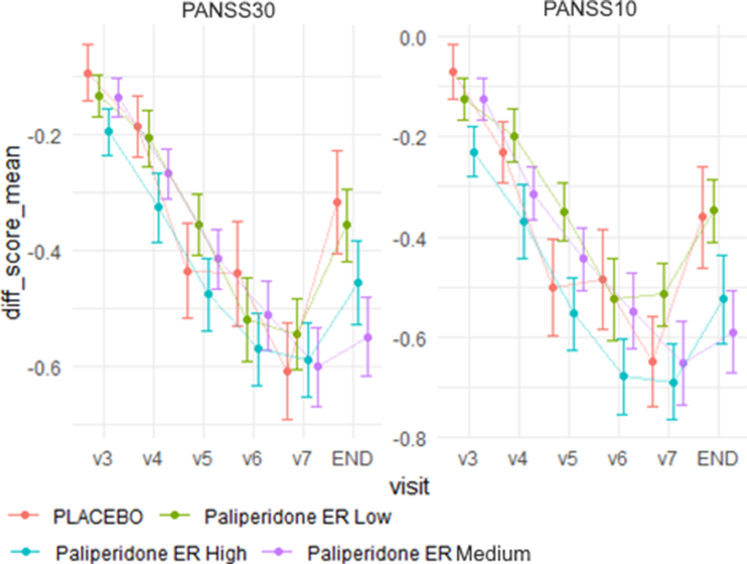# Replicating and Extending the Reliability, Criterion Validity, and Treatment Sensitivity of the PANSS10 and PANSS20 for Pediatric Trials

**DOI:** 10.1016/j.jaacop.2024.02.010

**Published:** 2024-05-23

**Authors:** Eric A. Youngstrom, Joshua A. Langfus, David Gordon Daniel, Joan Busner, Robert L. Findling

**Affiliations:** a Nationwide Children’s Hospital and The Ohio State University, Columbus, Ohio; b University of North Carolina, Chapel Hill, North Carolina; c Helping Give Away Psychological Science, Chapel Hill, North Carolina; d Signant Health, Blue Bell, Pennsylvania; e George Washington University, Washington, DC; f Virginia Commonwealth University, Richmond, Virginia

**Keywords:** psychosis, short form, psychopharmacology clinical trials, rating scale, children and adolescents

## Abstract

**Objective:**

Pediatric studies of schizophrenia have relied on the 30-item Positive and Negative Syndrome Scale (PANSS30) as a primary outcome measure. There have been many efforts to create shorter versions of it to reduce costs and burden. The present aim is to conduct a confirmatory investigation of the reliability and validity of 10- and 20-item abbreviated versions developed in a United States–based National Institute of Mental Health (NIMH) pediatric sample that reflects the 5-factor structure underlying the PANSS, adding more detailed examination of patient-level score reproducibility and extending the examination to a large, placebo-controlled, international pediatric trial.

**Method:**

We applied the same psychometric and treatment sensitivity analyses as in Findling *et al.* (2023) to an adolescent schizophrenia paliperidone randomized placebo-controlled trial (RCT), accessed via the Yale Open Data Access (YODA) secure data environment (described in Singh *et al.*, 2011). Analyses included confirmatory factor analyses, graded response models, ω reliability coefficients, tests of convergent criterion validity, sensitivity to change, and Bland–Altman plots to evaluate score reproducibility.

**Results:**

Using the paliperidone RCT dataset, with N = 201 participants between the ages of 12 and 17 years (mean age = 15.40, SD = 1.53 years; 59% male), the PANSS 10- or 20-item vs 30-item versions had similar average interitem correlations (0.11-0.15); ω^Total^ reliabilities of 0.78 to 0.89 with reliability >0.80 across patient presentations from mild residual symptoms to severe pathology; correlations of 0.92 and 0.98 with the 30-item total; partial eta-squared (η^2^) values for time, treatment, and time by treatment; and also correlations with Clinical Global Impression (CGI) severity and Children’s Global Assessment Scale (CGAS) ratings. Per-item scores differed by 0.04 points on average on the PANSS10 and by 0.01 points for the PANSS20 vs the PANSS30, all not significant.

**Conclusion:**

Results replicated reliability and validity findings for the PANSS10 and PANSS20 short forms in an international pediatric randomized placebo-controlled trial. Findings extend prior work by being the first to apply modern reliability models (ω) for multi-factor composites, also using Bland–Altman methods to evaluate patient-level score reproducibility. Scores based on the PANSS10 or PANSS20 reproduce traditional scores with high fidelity and low bias, offering substantial savings in terms of time, cost, and burden, especially when used for tracking progress or outcomes.

**Clinical guidance:**

• Consider using the 10-item or 20-item PANSS for regular monitoring and assessment of pediatric schizophrenia patients. These shorter versions maintain high reliability and can streamline the evaluation process, making it more efficient.

• Patient and family engagement: Use the abbreviated PANSS versions to engage more effectively with patients and their families. The reduced length of the assessment can alleviate the stress and fatigue associated with longer evaluations, potentially improving patient cooperation and the quality of the data collected.

## Plain language summary

A secondary analysis of data from a global clinical trial involving 201 young patients with schizophrenia found that shorter, 10 and 20-item versions of the standard 30-item schizophrenia assessment tool (PANSS) are equally reliable and valid. The abbreviated versions correlated >.9 with the full-scale scores, with no significant bias when comparing scores. This finding suggests that mental health assessments for young patients can be significantly shortened without losing accuracy, greatly reducing the time and effort required from both families and clinicians. This advancement in psychiatric evaluation could make mental health care less burdensome for families dealing with schizophrenia.

The Positive and Negative Syndrome Scale (PANSS)^1^ has become the standard outcome measure in trials and studies investigating psychotic symptoms in adolescents. It is an accepted primary outcome measure for filings with the Food and Drug Administration and other regulatory agencies, and it has been the primary outcome used in pediatric schizophrenia trials funded by the National Institute of Mental Health (NIMH) as well as pharmaceutical companies.

The PANSS has remained essentially unchanged since it was written in the 1980s. The selection of items and the organization of items into subscales were based on clinical observation rather than the modern statistical analytic methods that are now considered standard of practice for scale design.^2^ For example, there were no exploratory or confirmatory factor analyses guiding the organization of items into subscales. Furthermore, the items were written for adult patients.

Some additional practical considerations are that scoring the PANSS uses interviewer judgment to combine patient response, informant response, and direct observation on 30 items. The interviews can be lengthy, and they require a substantial investment in interviewer training, calibration of scoring across raters, and monitoring procedures to maintain accuracy and precision of symptom severity measurement throughout a clinical trial to avoid rater drift. Inadequate training and monitoring increases error in ratings, potentially leading to enrollment of inappropriate cases, inflated placebo response, and/or underestimated treatment effects.

To address these issues, research has applied increasingly sophisticated statistical models and prioritized exploration of shorter forms of the PANSS in adults^3^, ^4^, ^5^, ^6^, ^7^, ^8^ and, more recently, in pediatric samples.^9^^,^^10^ These could reduce interview length by eliminating weaker items while keeping the reliability and validity high. Consensus is that the PANSS has 5 underlying, modestly correlated factors rather than a single dominant underlying factor.^8^ Thus it would be more appropriate to treat the PANSS as a composite measure, much like a personality inventory that measures multiple dimensions, rather than concentrating on a single total score that blends all 5 constructs together into 1 single rating.^11^

A second point of consensus is that some items are poor from a statistical perspective across samples. The items cover general psychopathology as well as positive and negative symptoms of schizophrenia. Many of these items do not correlate highly with the others, and do not load on a major factor. For example, items about lack of judgment and insight (item G12) and disorientation (G10) have not been retained in any published short form. Their poor performance could be due to difficulty in rating via interview, the patient misunderstanding or misinterpreting the item, or empirically low validity.^12^ Item heterogeneity also means that some could be asking about associated features or comorbid conditions vs core syndrome features.

Given these 2 points of consensus—guided by findings with adult samples^3^, ^4^, ^5^, ^6^, ^7^, ^8^ as well as a pediatric one,^10^ it should be possible to develop short forms that can retain strong reliability, especially by eliminating weak items while also showing a strong correlation with the full-length version. When a measure has distinct factors, short forms need to keep the same factor structure to preserve the content coverage of the longer form.^13^ This seems especially pertinent in the case of the PANSS: several studies in adults have shown that the factors have differential sensitivity to treatment effects.^5^^,^^8^ If the full-length PANSS measures 5 distinct things that do not respond equally well to a treatment, short forms that omit some of the 5 factors will provide misleading impressions about the efficacy of the treatment. Of note, only 1 of the prior short forms published with the PANSS has retained all 5 of the subscales suggested by the factor analysis, and it has been evaluated only in adults.^7^

Considering the promise of developing an abbreviated PANSS in adults, our group explored whether a shortened PANSS could be developed for use in pediatric-aged populations. The current nosology uses the same criteria for schizophrenia in children and adolescents as well as adults,^14^ and scales such as the PANSS have been used with pediatric samples without developmental modification. Using data from the NIMH-funded Treatment of Early-Onset Schizophrenia Spectrum Disorders (TEOSS) pediatric trial,^15^ we made 2 short forms of the PANSS using a combination of item response theory and factor analysis. To our knowledge, these were the first short versions to measure all 5 factors underlying the PANSS for a pediatric age group. A series of analyses (briefly described in the Methods, below), a 10-item version (PANSS10) that kept the 2 best items for each of the 5 factors, and a 20-item version (PANSS20) added items to the 5 scales.^10^ These forms showed excellent model fit, strong reliability across a broad range of symptom severity, and good coverage of the constructs assessed by the full-length PANSS. Despite these promising results, replication in an independent sample with both different interviewers and treatment intervention would provide important information about the generalizability of the performance of these abbreviated versions.^2^^,^^16^

The goal of the present study was to perform a secondary analysis of another large pediatric schizophrenia clinical trial to compare the performance of the PANSS10 and PANSS20 to the full-length PANSS30. We secured access to a sample investigating a different compound (paliperidone) in a double-masked trial with a placebo arm,^17^ whereas TEOSS had no placebo arm. Specific aims for the present study include evaluating the performance of the PANSS10 and 20-item short forms compared to the full-length 30-item version in terms of statistical model fit, reliability of the subscale scores, content coverage, and calibration against the full-length version, and comparison of convergent validity with secondary outcome measures of functioning and treatment response. We also evaluated the sensitivity of the scales to time, treatment, and time by treatment effects, and compared the effect sizes to those generated by the full-length version. In addition, we reported precision and change benchmarks^10^^,^^18^ that will facilitate application to individual clinical cases.

## Method

### Measures

#### Positive and Negative Syndrome Scales

The Positive and Negative Syndrome Scales (PANSS)^1^ is a 30-item instrument rating positive (P), negative (N), and general psychopathology (G) cognitive and affective symptoms often associated with schizophrenia and psychosis using a scale of 1 (absent) to 7 (extreme). Highly trained raters (qualified investigators who achieved a high interrater reliability coefficient [inter-rater intracclass correlation coefficient = 0.874^17^]) scored each item at each study visit based on direct interview of the youth as well as the primary caregiver, asking about the past week. Present analyses estimated both a total score (traditionally used as the primary outcome measure) and 5 subscales based on the analyses in Findling *et al.*^10^ using PANSS10 and 20-item versions. The prior study’s item selection followed several steps, beginning with exploratory factor analysis using a suite of the most accurate methods to determine the optimal number of factors,^19^^,^^20^ followed by examination of the item loadings on their factors in a rotated principal axis factoring solution. The 5-factor solution that was statistically preferable also aligned with the dominant model reported in prior studies with adult data. A confirmatory factor analysis (CFA) evaluated the 5-factor model retaining the strongest-loading items with no cross loadings, using *k*-fold cross-validation to avoid overfitting. After unidimensionality tests, graded response models were used to evaluate items, and reliability coefficients and several convergent correlations were used to examine validity.

We used the models from Findling *et al.*^10^ as our *a priori* models, and evaluated their performance in a confirmatory framework.^21^ Present results used the item average rather than sum, and some applications of the PANSS use a format of 0 to 6 instead of 1 to 7 for scaling the items. These will produce identical results in terms of statistical significance, reliability, correlations, and slopes. Changing scaling does affect regression intercepts and clinical benchmarks, such as rubrics for “mild,” “moderate,” or “severe” scores. Using item averages made it easier to compare agreement and calibration of scales with different numbers of items.^11^ We refer to the total composite scores from each scale as the PANSS10, PANSS20, and PANSS30 in the Results and tables.

#### Criterion Validity Measures

Study raters also completed the Clinical Global Impressions of Severity (CGI-S) at each visit, with ratings from 1 (normal, not at all ill) to 7 (among the most extremely ill patients),^22^ along with the Clinical Global Assessment Scale (CGAS).^23^ The CGI-S and CGAS were chosen because they are well known, widely used in clinical trials, and accepted as primary or secondary outcome measures by most regulatory bodies, including the US Food and Drug Administration and corresponding bodies in Europe.

### Statistical Analyses

Based on the combination of prior work in an independent sample, as well as the congruence with adult findings, we shifted to a more strictly confirmatory framework in the present study.^21^ Thus, we modified the analytic plan used in Findling *et al.*^10^ by dropping the exploratory factor analyses, using the 5-factor models that were the product of the prior paper as the *a priori* models for the CFAs in the present paper. We also added analyses to examine the calibration and bias of the short-form vs long-form scores (detailed below).

We ran descriptive statistics and compared demographics and results to those reported in the primary outcome paper.^17^ We used confirmatory factor analysis (CFA) with ML estimation to evaluate fit of the 10-item and 20-item 5-factor/subscale models developed in Findling *et al.*,^10^ as well as the 30-item 5-factor model recommended based on Marder *et al.*^8^ We used average item correlation as an estimate of internal consistency not dependent on scale length, Guttman λ^6^ as a second estimate that does not assume a single underlying factor (a well-known limitation of Cronbach alpha),^11^ and then item response theory (IRT) to estimate marginal reliability across a range of severity levels, as well as option characteristics for each item.^24^ The clinical value added by the IRT model includes showing whether the precision of scores decreases at mild or very severe levels of pathology. Because the PANSS measures more than 2 factor, we reported 3 versions of ω.^11^ ω^Total^ combines reliable variance from a general factor and the 5 specific factors. Two alternative versions parse the reliability of the total composite score into reliable variance attributable to the general factor (ω^Hierarchical^), and the variance in the total score that reflects reliable information about the 5 specific factors (delusions, withdrawn/apathetic, etc; ω^Specific^).

Content coverage and calibration were estimated by regressing the 30-item score on each short form, as well as using Bland–Altman plots to show patterns in agreement.^25^ These plots look at agreement between scores based on the full-length vs the short forms, and examine both average bias and whether discrepancies tend to increase at low or high score ranges.

Criterion validity looked at within-visit correlation between the 30-, 20-, and 10-item scores and the CGI-S and CGAS, both at baseline and across all visits, with the Steiger test of dependent correlations testing for differences in validity coefficients. Treatment effects were estimated using partial η^2^ for time, treatment arm, and time by treatment interaction via generalized linear models (GLM).

### Procedure

We prepared a secondary analysis request to YODA. After approval from the UNC IRB and YODA, the analysis team (JAL, EAY) received secure logins and accessed the data for analysis. Details about the conduct of the clinical trial itself are reported in Singh *et al.*^17^ Briefly, the protocol was a 6-week, double-masked, 4-arm study that used (30-item) PANSS total scores between 60 and 120 as an entry criterion. Subjects were randomly assigned to placebo or to 1 of 3 daily doses of paliperidone extended release (ER): 1.5 mg (low), 3 mg (medium), or 6 mg (high) for subjects weighing <51 kg, and 1.5 mg (low), 6 mg (medium), or 12 mg (high) for subjects weighing ≥51 kg at entry. The primary outcome was change in PANSS total score from baseline to day 43 or last post-baseline assessment if discontinuing earlier.

## Results

### Participants

A total of 201 participants between the ages of 12 and 17 years (mean age = 15.40 years, SD = 1.53 years; 59% male), drawing from 35 treatment centers in 5 countries, entered the randomized acute treatment phase. Our analyses of baseline data used N = 201, whereas some supplemental psychometric analyses used either all observed visits or last observation carried forward (LOCF), as indicated.

### CFA of 5-Factor Models Using 30, 20, and 10 Items

Table S1 (available online) reports fit indices for the 5-factor model using each proposed set of items. Because the item indicators are different, these are not “nested” models, so direct testing of comparative model fit is not possible. It should be noted that we fixed loadings for estimation consistently with Findling *et al.*, except that for the 30-item set we used the Marder *et al.* model, because in the TEOSS data 4 of the items failed to load on any factor in exploratory or confirmatory models.

The 5-factor models performed much better than single factor models in all 3 item sets (Table S1, available online). In the 30-item set, the Marder model showed poor fit across all indices. One item failed to load even *p* < .05 on the target factor (N07, stereotypical thinking), and several others showed only modest primary loadings. In contrast, the 10- and 20-item 5-factor models showed adequate fit (Table S1), with all items loading substantially on the correct factors.

### Reliability and Precision of the Short Forms

Table 1 shows the reliability and precision estimates for the composite scores. The average inter-item correlation was 0.11 for the 10 items, 0.12 for the 30 items, and 0.15 for the 20 items. Ω^Total^, the most conceptually appropriate reliability estimate given that the PANSS composites measure 5 modestly correlated factors, ranged from 0.78 (PANSS10) to 0.89 (PANSS30). All ω coefficients are reported in Table 2.

**Table 1:** Criterion Correlations for Full-Length, PANSS10, and 20-Item Pediatric PANSS Short Forms (N = 201, N = 1,037 Observations for Change From Baseline Eta-Squared [η^2^])

Scale	Baseline criterion correlationsBaseline criterion correlations			η^2^ for LOCF analysesη^2^ for LOCF analyses		
	PANSS30 total	CGI-severity	CGAS	Change from baseline	Between treatment arms	Change by treatment arms
PANSS30	1.00	0.62^†^	–0.56^†^	0.17^†^	0.00	0.00
PANSS10	0.86^†^	0.53^†^	–0.47^†^	0.18^†^	0.00	0.00
PANSS20	0.97^†^	0.59^†^	–0.54^†^	0.15^†^	0.00	0.00
Two-item scores						
Aggression (poor aggressive impulse control; hostility)	0.39^†^	0.18∗	–0.10 (ns)	0.08	0.03	0.00
Withdrawal/apathy (emotional withdrawal; passive/apathetic social withdrawal)	0.53^†^	0.36^†^	–0.44^†^	0.06	0.01	0.00
Thought disturbance (conceptual disorganization; poor attention)	0.61^†^	0.30^†^	–0.36^†^	0.07	0.02	0.00
Internalizing (anxiety; guilt feelings)	0.33^†^	0.37^†^	–0.27∗∗∗	0.06	0.00	0.00
Delusions/odd content (delusions; unusual thought content)	0.41^†^	0.29^†^	–0.21∗∗	0.12	0.01	0.00

Note: Items for each factor-based subscale are listed in parentheses. CGI = Clinical Global Impressions; CGAS = Child Global Assessment Scale; LOCF = last observation carried forward.

∗*p* < .05; ∗∗*p* < .005; ∗∗∗*p* < .0005; ^**†**^*p* < .00005; n.s. *p* > .05 2-tailed, unless otherwise indicated.

**Table 2:** Reliability, Correlation With Full-Length Scale, and Length Reduction for Composite Scores Using Baseline Data From Acute Phase (N = 201)

Version	PANSS10	20-Item	Full-length
Mean	3.08	3.05	3.04
SD	0.47	0.47	0.43
Range	2.00 to 4.30	2.05 to 4.25	2.10 to 3.97
ω^Total^	0.78	0.87	0.89
ω^Hierarchical^^(higher order factor, Schmid-Leiman)^	0.65	0.73	0.74
ω^Specific^^(variance due to 5 factors)^	0.38	0.22	0.23
Observed mean inter-item correlation	0.11	0.15	0.12
Observed λ^6^	0.78	0.86	0.89
Projected correlation with full	0.72	0.81	—
Observed correlation	0.92	0.98	—
Reliability >0.8 across range (IRT θ levels)	–1.5 to 4.1	–3.3 to 5.0	–3.6 to 5.8
Discrepancy (Short – Long) in points	0.05	0.02	—
SD of discrepancy	0.24	0.12	—
95% limits of agreement	–0.42 to 0.51	–0.21 to 0.24	—
Savings in length (%)	67	33	0
Standard error of measurement	0.22	0.17	0.14
Standard drror of difference	0.31	0.24	0.20
90% Critical change	0.51	0.39	0.33
95% Critical change	0.61	0.47	0.40
Minimal important difference (*d* = ∼0.5)	0.24	0.24	0.22

Note: Observed correlations are based on embedded item administration. Standard errors used ω^Total^ as reliability. The 90% and 95% critical change values are benchmarks for change thresholds that reflect “reliable change” rather than score fluctuations for reasons besides treatment response (adapted from the Jacobson Reliable Change Index^18^). Minimal important difference is a benchmark for the smallest amount of change that is likely to be considered meaningful by a patient or family.

We also examined the reliability coefficients in all available observations. The reliability estimates all increased considerably over time (eg, average inter-item correlations of 0.25-0.27), reflecting the increased variability in scores as patients received the assigned treatment, as well as potential restriction of range at study entry, as cases needed to have PANSS30 raw totals of between 60 and 120 to enter the acute treatment phase. All coefficients were larger than those derived from the baseline data due to the increased variance in scores over the course of treatment, which is a well-established artifact.^18^^,^^26^

**Figure 1:**
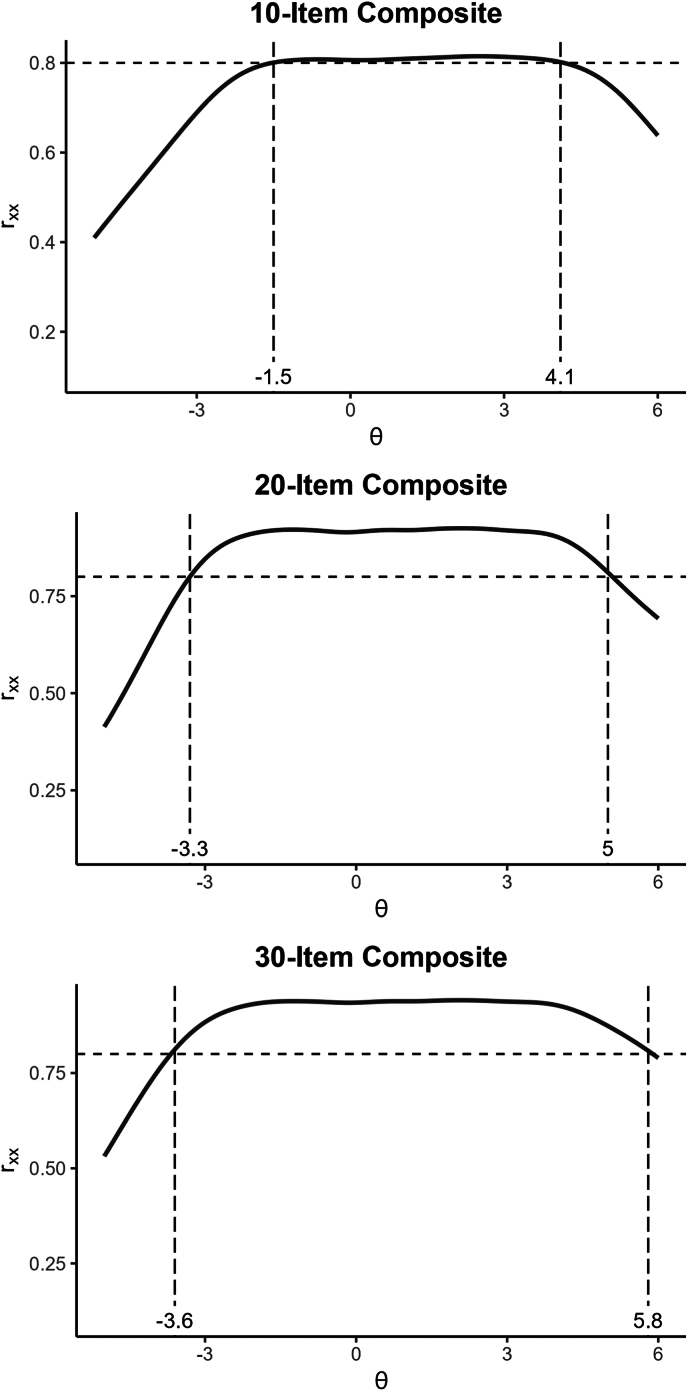
Reliability Coverage of Composite Scores Based on Graded Response Model (N = 201)

**Figure 2:**
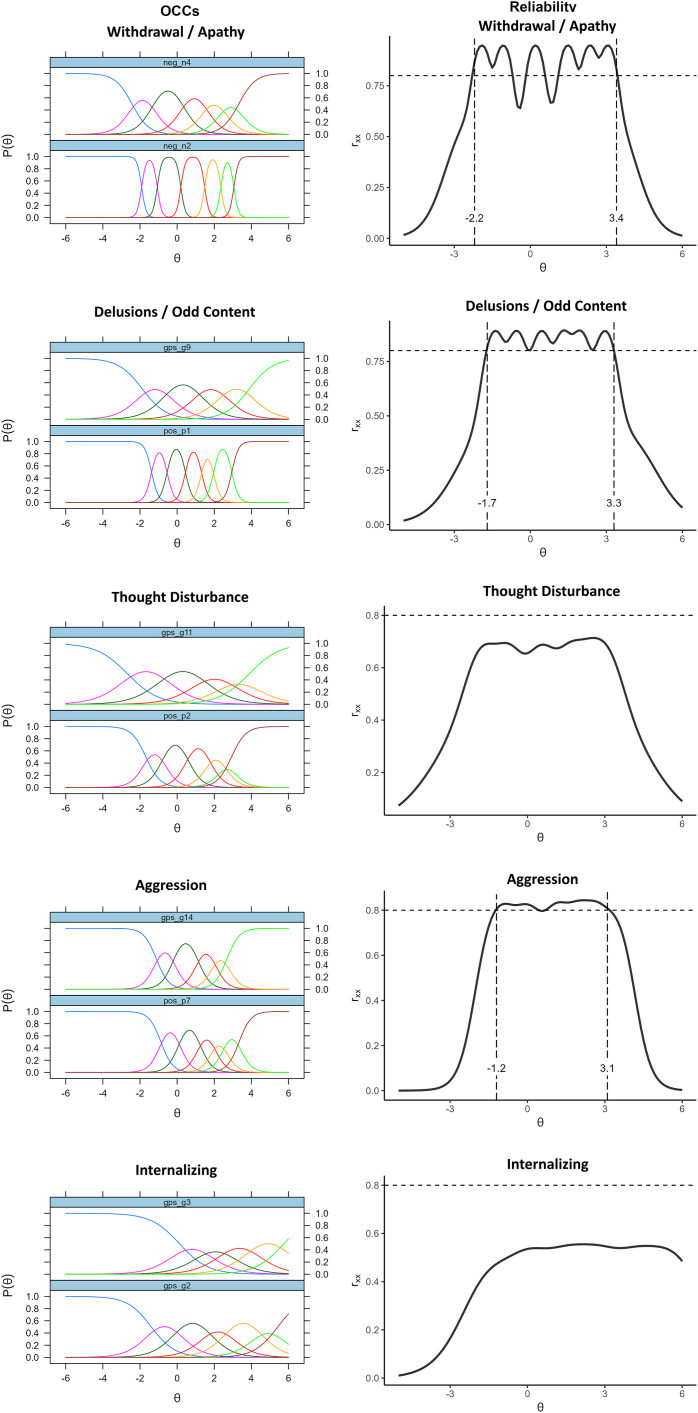
Information and Reliability Estimates From Item Response Theory Analysis of Short Forms^17^ (N = 201) ***Note:**Adapted from Youngstrom *et al*.*^27^ (https://doi.org/10.17605/OSF.IO/T5MSZ; published under Creative Commons license CC BY: https://creativecommons.org/ licenses/by/4.0).

IRT analyses showed that the PANSS10 composite had reliability >0.80 between theta levels of −1.5 to +4.1 SDs above the average trait level (Figure 1); so did all of the 2-item short facets except for the cognitive and internalizing scales (Figure 2^27^). The PANSS20 form had reliability >0.80 over an even wider range of −3.3 to +5.0. The option characteristic curves looked good for items, and very good for the short forms (which focused on single factors), although the highest response options were rarely selected for a few of the items in this largely outpatient sample. Detailed item option characteristics are available in Table S2, available online. In contrast, IRT analyses of the PANSS30 items found many items with flat information curves (eg, G1, G3, P5, G10) and implausible parameter estimates (consistent with the poor loadings in the factor analyses). The IRT-estimated reliability for the PANSS30 total was >0.80 from theta −3.6 to 5.8, negligibly different from the PANSS20 version.

Table 1 also includes the standard errors of measurement (SEM) and the difference score (SEd) for 2 administrations of the same form, and critical values for 90% and 95% confidence differences between the same patient’s score at 2 time points reflects “reliable change,”^28^ along with a benchmark for “minimally important difference” (MID), an estimate of the smallest change likely to be considered clinically meaningful.^2^

### Content Coverage and Accuracy of Short Forms

**Figure 3:**
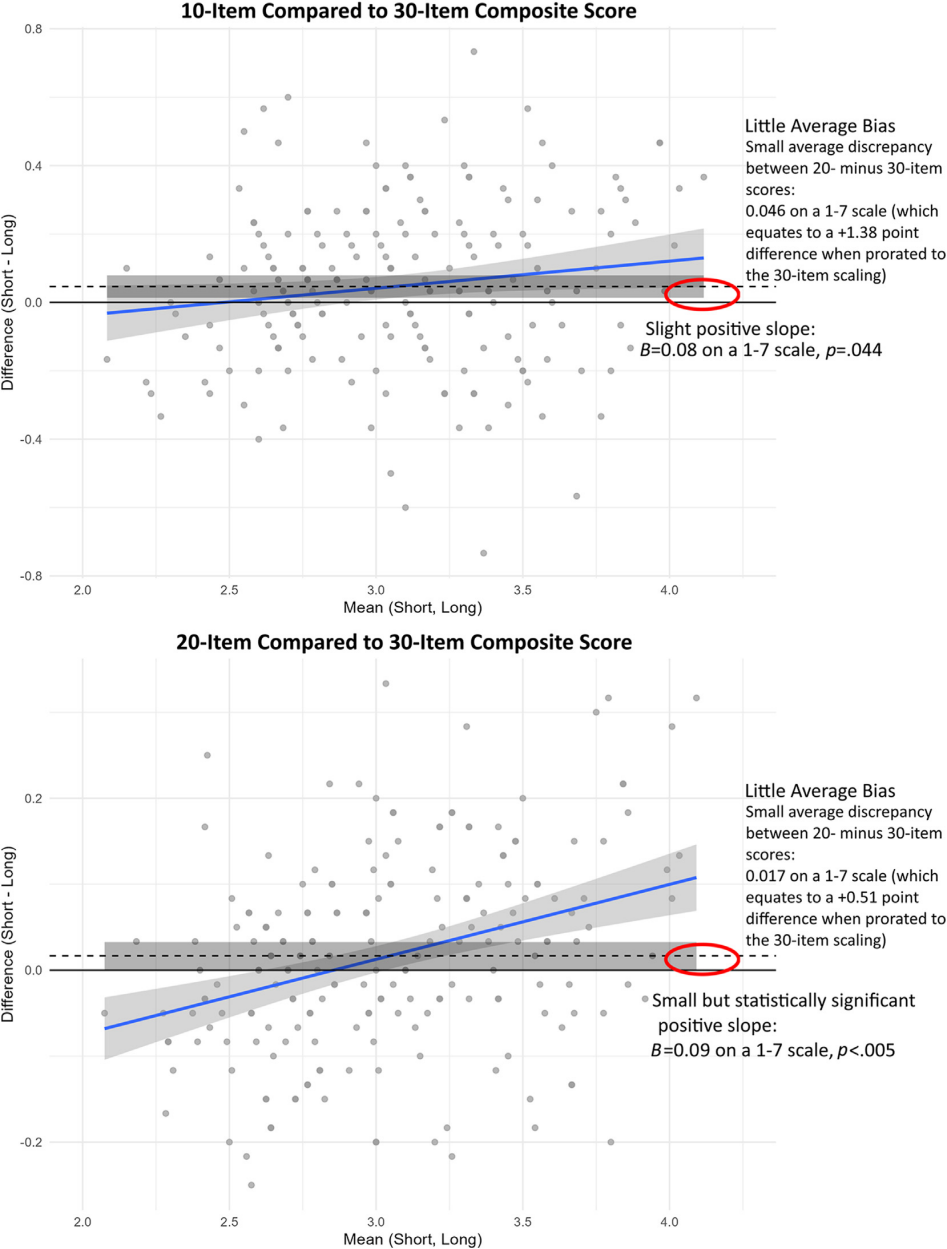
Bland**–**Altman Plots Comparing Accuracy of PANSS10, PANSS20, and PANSS30 Scores ***Note:****Dashed line indicates average bias; blue line indicates regression looking for tendency to over- or underestimate scores compared to the original version. A positive discrepancy indicates that the short form produces a higher score estimate. PANSS = Positive and Negative Syndrome Scale*.

Content coverage was *r* = 0.86 for the PANSS10 and 0.97 for the PANSS20 with the full-length scale using the baseline scores, and *r* = 0.92 for the PANSS10 and 0.98 for the PANSS20 with the full-length scale based on all observations across all waves (all *p* < .00005). Both were larger than the projected correlations based on the internal consistency and reduced scale length (*r*^*hat*^ = 0.72 for a PANSS10 version and 0.81 for the PANSS20 using the more conservative baseline data). The Bland–Altman plots indicated a slight tendency for scores to be higher on the short form than on the full-length form as scores increased; however, the average discrepancy was negligible (ie, <0.05 points). Conveniently, it was smallest in the score range used as an enrollment criterion for the trial (eg, average discrepancy of zero at observed scores around an item average of 2.0, or a PANSS30 raw score of 60) (Figure 3).

### Criterion Validity of the Short Forms: Convergent Correlations

Table 1 presents convergent correlations for the original 30-item, the 10- and 20-item short forms, the CGI-S, and the C-GAS. The PANSS10 form correlated 0.86 with the full-length form. It converged *r* = 0.53 with the CGI-S (vs 0.62 for the 30-item and CGI-S); this difference was small albeit statistically significant given the very high correlation between the 2 PANSS versions (*t* = 3.05, *p* = .003). Similarly, the CGAS correlations were 0.47 with the PANSS10 vs 0.56 with the original, also significantly different per the Steiger test (*t* = 2.89, *p* = .004). Table 1 includes the convergent correlations for the PANSS20 form as well, which were even closer in matching the PANSS30 (*t* = 2.20, *p* = .029 for the difference in CGI-S correlations, and *t* = 1.39, *p* = .167 for the CGAS).

### Sensitivity to Treatment Effects

**Figure S1:**
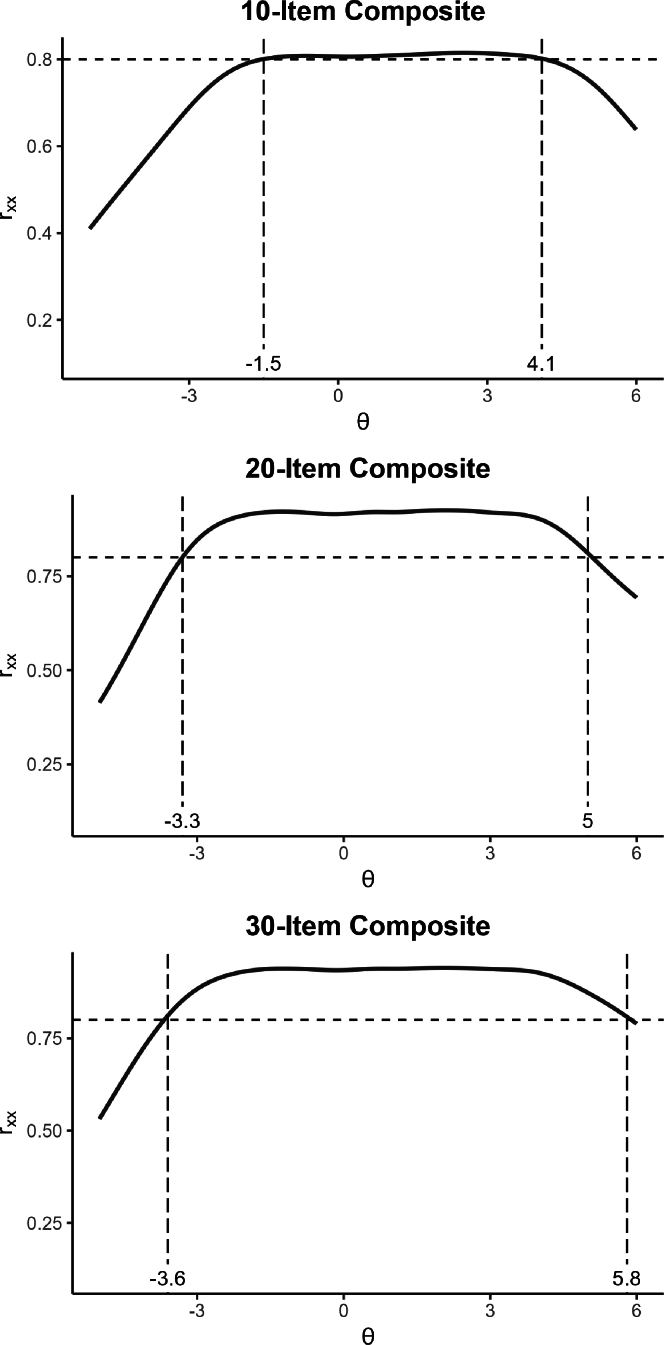


The PANSS10, PANSS20, and PANSS30 composites produced essentially identical estimates of treatment effects based on several analyses. The eta-squared (η^2^) value for time (comparing baseline to end of phase) ranged from 0.15 (based on the 20-item) and 0.18 (PANSS10), all highly significant, indicating moderate to large improvement over time. The main effects for treatment, as well as the time-by-treatment interaction, were not significant (η^2^ < 0.01). These were 3 df tests pooling effects across all treatment arms. The original report found separation between only 1 treatment arm (the medium dose) and placebo. Figure S1 (available online) plots the means and standard errors for all 4 treatment arms using both the 10- and 30-item scales, showing highly consistent results. Duplicating the models reported in Singh *et al.*^17^ as closely as possible based on documentation also produced consistent results with the PANSS10, PANSS20, and PANSS30 (Table S3, available online). CGI severity at last visit correlated *r* = 0.51 (CI = 0.37-0.64) with change from baseline to end of phase measured via the 30-item version, and 0.50 with the PANSS10 and PANSS20 (CI = 0.35-0.62 for both) versions. Additional supplemental tables (Tables S4-S10, available online) provide detailed information about scale correlations, discrepancy scores, and reliability and precision statistics.

## Discussion

The goal of the present study was to replicate and extend investigation of the psychometrics of short forms of the PANSS developed in 1 randomized treatment study of pediatric patients with schizophrenia (NIMH TEOSS study^15^) by testing them in a wholly independent randomized study of pediatric patients with schizophrenia (paliperidone study^17^). The present results show that 10- and 20-item short forms originally built in the TEOSS sample replicated well in this new dataset. Specifically, the total scores from both total composite scores achieved good reliability and extremely high correlation with scores obtained using the standard 30-item version. The short forms also showed no evidence of clinical bias based on Bland–Altman plots and regression analyses. Prorated scores based on the short forms were essentially identical to those based on the full-length form, especially around the score range that is typically targeted for enrollment in clinical trials (eg, total scores of 60-120). Short-form scores and full-length scores showed correlations that were similar to those of the CGAS and CGI-S scores also, and similar sensitivity to treatment effects based on mixed regression analysis, visual inspection of week-by-week data, and post hoc analyses.

The present results also confirmed prior findings that the PANSS items reflect multiple constructs with low correlation, consistent with findings in multiple adult studies.^3^, ^4^, ^5^, ^6^, ^7^, ^8^ We used CFA and graded response IRT models to fit the 5-factor structure reported previously by Findling *et al.*,^10^ which also aligned with the consensus model in adult studies.^6^^,^^8^ Whether using 30, 20, or 10 items, the 5-factor model fit markedly better than a 1-factor model, although the adequacy of the fit was only moderate for any of the 5-factor models. IRT analyses showed that the total composite score provided high reliability across an extremely wide range of severity levels. For clinical trials and patient samples, the range of interest would extend from mild or asymptomatic levels (eg, 1.5-2 SD below “moderate” pathology) to highly impaired (eg, 2.5 or more SD above “moderate”). All 3 scales exceeded this range of coverage, indicating that the scales would provide similarly precise estimates of change in severe, moderate, or mild ranges of severity. Items assessing mannerisms and posturing (P5), stereotypical thinking (N7), and lack of insight (G12) showed weak factor loadings and poor item characteristics, also consistent with prior work in adult as well as pediatric samples.

Reliability analyses were consistent with there being multiple underlying factors. We estimated 3 ω coefficients, which estimate the total reliability of the scale (ω_Total_), as well as dividing that reliable variance into pieces reflecting a global factor (ω_Hierarchical_) vs variance due to specific underlying factors (ω_Specific)_. The preferred index of overall reliability for a multifactor composite is Ω_Total_, not Cronbach α.^11^ High values of ω_Hierarchical_ would support emphasizing a total score, as is the case with many measures of cognitive functioning. In contrast, relatively large ω_Specific_ coefficients would motivate emphasizing the subscales or treating them as separate scales entirely,^29^ as is the case with many measures of personality. For the PANSS, ω_Total_ was much bigger than the ω_Hierarchical_, consistent with the multi-factor structure.

The findings for the 5 subscales were more mixed. Three of them showed good reliability even in their 2 item versions, although the thought disturbance scale and the internalizing scale did not appear as reliable in the present data. The ω_Specific_ values also varied, with some rising above the suggested rule of thumb for prioritizing the subscale, and others falling below it. Consistent with prior findings in adult studies, the 5 subscales showed distinct patterns of response to the intervention. Low reliability does not appear to be a complete explanation for the results in the present data, as the aggression scale showed the lowest correlations with CGI-S and CGAS despite higher reliability, and the thought disturbance and internalizing scales showed higher correlations. More investigation of the validity and treatment sensitivity of the subscales is warranted.

To our knowledge, this is the first study to use contemporary models of reliability such as the ω coefficients with any sample (pediatric or adult) using the PANSS. This is a significant advance, given that studies consistently show that the PANSS has a multi-factor structure and not a single dominant factor, as would be assumed when using the Cronbach α. Modeling the multifactor structure not only provides a more accurate reliability estimate for the total composite score but also informs decisions about which subscales are more reliable and thus likelier to detect differential treatment effects.

This also is the first paper to use Bland–Altman plots^25^ to examine the accuracy of scores based on the short forms vs the traditional 30-item version. Bland-Altman plots are widely used in chemistry, pharmacology, and other areas of medicine,^30^ although they are less well known in psychiatry and psychology. They provide easily interpretable estimates of score bias as well as the “limits of agreement.” Results here indicate that the short forms could be used instead of the 30-item version for screening, baseline evaluation, or tracking of change over time with a high degree of interchangeability.

Several factors accentuate confidence in findings, including use of a completely independent sample with different raters, different families, and a different treatment compound.^16^^,^^31^ In addition, the PANSS and other interview components were translated into multiple languages for completion of the original trial. Each of these factors adds heterogeneity to the data and increases the external validity and generalizability of the results.

Limitations of the present paper include that it is a secondary analysis, and that the PANSS was administered in a complete 30-item format. From a technical perspective, checking the reliability and validity of the 10- and 20-item versions when not embedded in the full-length interview would verify that context effects do not change item performance. The reduced burden and length of a shortened interview might also increase rater and participant focus, further improving the reliability of the scales. Another limitation is that we could not exactly replicate some of the original analyses reported in Singh *et al.*,^17^ as they were not described in sufficient detail in the published report. Additional research also would be valuable for investigating the reliability and treatment sensitivity of the 5 subscales complied by the factor models.

Current findings combine with the body of evidence^3^, ^4^, ^5^, ^6^, ^7^, ^8^^,^^10^ to suggest 4 tentative recommendations:

(1) Phase out the 30-item version of the PANSS, which includes multiple items that consistently have performed poorly.^6^^,^^10^ Dropping them would streamline the interview and improve the assessment signal by reducing construct irrelevant variances.
(2) Consider using the PANSS10 or PANSS20 in pediatric samples. The PANSS10 may be ideal for situations in which time is paramount, such as intensive assessment during clinical trials or many clinical practice settings. The longer PANSS20 offers even higher content coverage while removing the weak items, making it appealing for well-resourced clinical trials. Results also suggest the potential for development of an entirely new instrument with more and stronger indicators per factor. However, new measure construction would trade potential improvements in reliability and coverage for losses in terms comparability with work using the PANSS. It also would take time to achieve recognition and acceptance by reviewers, funders, and regulatory agencies. Optimization by careful subtraction offers a balance between improvements vs recognition and comparability.
(3) Reconceptualize the total score as a composite summarizing the patient status across 5 dimensions of psychopathology, not as a single construct.^11^
(4) The 5 subscales could be interesting to explore as secondary outcomes in pediatric patients. Present and prior analyses indicate that treatments have varying effects on the different dimensions. Reliance on a total score obscures this: combining all of these into a single score may be akin to summing the Big Five dimensions into a single score of “total personality.”^3^ In clinical practice, having data about recalcitrant symptom dimensions may treatment inform choices.

Future research should focus on differential treatment sensitivity and other aspects of validity, particularly for the subscales. It would be intriguing to look for different subscale profiles of treatment response to varying interventions, much as cognitive–behavioral therapy and pharmacotherapy produce different patterns of response in depressive symptomatology.^32^ Another interesting question is whether the PANSS items and scales show measurement invariance across age cohorts. Although current nosology uses the same criteria for psychosis and schizophrenia regardless of age, and the 5-factor structure also appears to be largely consistent, there may be neurocognitive, hormonal, and social processes that affect score patterns with maturation. Even pending these investigations, the near-unanimity of findings about the 5-factor structure and weak items on the 30-item set offer opportunities to improve assessment now.

## CRediT authorship contribution statement

**Eric A. Youngstrom:** Writing – review & editing, Writing – original draft, Formal analysis, Conceptualization. **Joshua A. Langfus:** Writing – review & editing, Software, Formal analysis. **David Gordon Daniel:** Writing – review & editing, Conceptualization. **Joan Busner:** Writing – review & editing, Data curation, Conceptualization. **Robert L. Findling:** Writing – review & editing, Conceptualization.

## Figures and Tables

**Figure S2:**